# Reducing paralytic ileus after radical cystectomy: The role of advanced haemodynamic monitoring

**DOI:** 10.1002/bco2.70232

**Published:** 2026-08-24

**Authors:** Constantin Rieger, Till Löhr, Julian Heidenreich, Anna Rieger, Alexander Mathes, David Sander, Florian A. Schmid, Christian Bach, David Pfister, Axel Heidenreich

**Affiliations:** ^1^ Department of Urology, Urologic Oncology, Robot‐Assisted and Specialized Urologic Surgery University of Cologne Cologne Germany; ^2^ Department of Anesthesiology and Intensive Care Medicine University Hospital of Cologne Cologne Germany; ^3^ Department of Urology University Hospital of Zurich Zurich Switzerland; ^4^ Department of Urology Medical University Vienna Vienna Austria

**Keywords:** FloTrac® system, hemodynamic monitoring, postoperative gastrointestinal recovery, postoperative paralytic ileus, radical cystectomy

## Abstract

**Objective:**

Despite highly standardized surgical procedures, approximately 20%–30% of patients develop postoperative paralytic ileus following radical cystectomy, representing the highest incidence among abdominopelvic surgeries. The objective of this study was to identify additional predictors of paralytic ileus in a large single‐centre cohort. Particular emphasis was placed on expanding the evidence regarding the effectiveness of advanced hemodynamic monitoring using the FloTrac® system.

**Patients and methods:**

We retrospectively analysed 383 patients and finally included 296 patients who underwent open or robotic radical cystectomy between 2018 and 2024 at our centre. Of these, 49 patients (16.6%) received intraoperative, minimal invasive FloTrac® monitoring. Perioperative variables were compared between groups, and predictors of paralytic ileus were determined using univariable and multivariable logistic regression analysis.

**Results:**

Paralytic ileus occurred in 23.0% of patients overall, with a significantly lower incidence in the FloTrac® group (10.2% vs. 21.3%), corresponding to a 60% relative risk reduction. In multivariable analysis, FloTrac® monitoring (OR 0.37, 95% CI 0.18–0.78, *p* = 0.008), female sex (OR 3.78, 95% CI 1.83–7.81, *p* < 0.001) and higher body weight (OR 1.04, 95% CI 1.00–1.08, *p* = 0.034) emerged as independent predictors. FloTrac® monitoring was further associated with reduced intraoperative blood loss and lower colloid infusion volumes. All other peri‐operative and postoperative variables, including complication rates (Clavien‐Dindo ≥ 3), did not differ significantly between FloTrac® and control group (*p* ≥ 0.05).

**Conclusions:**

FloTrac® monitoring was independently associated with a markedly lower risk of postoperative paralytic ileus after radical cystectomy. Female sex and higher body weight were identified as additional risk factors. Our findings underscore the potential of FloTrac®‐Monitoring to enhance postoperative gastrointestinal recovery for radical cystectomy. Nevertheless, overall surgical complication rates remained comparable.

## 1 INTRODUCTION

Radical cystectomy represents the international gold standard for the treatment of muscle‐invasive urothelial carcinoma (MIBC) of the bladder.^1^ Additional extended indications include curative intent in cases of bacillus Calmette–Guérin (BCG)‐refractory nonmuscle‐invasive bladder cancer (NMIBC), as well as palliative indications such as symptomatic, locally advanced prostate cancer.^2^

Despite highly standardized surgical procedures, approximately 20%–30% of patients develop a postoperative paralytic ileus, which represents the highest incidence among oncological abdominopelvic surgeries.^3^, ^4^, ^5^ Paralytic ileus, accompanied by abdominal distension, recurrent nausea and vomiting, can trigger secondary complications such as fascial dehiscence, which may necessitate reoperations.^6^ These secondary interventions can, in turn, predispose patients to long‐term complications, such as ureteral strictures. Furthermore, hospital stays are significantly prolonged, and healthcare costs may be nearly doubled, emphasizing the high socioeconomic burden of this complication.^7^

Several retrospective studies have investigated potential predictors and risk factors for paralytic ileus. Among the identified factors, a history of prior abdominal surgery and increased perioperative fluid administration have been shown to increase the risk of developing postoperative ileus.^8^

To optimize perioperative fluid management and thereby reduce the incidence of paralytic ileus, intraoperative goal directed fluid therapy—particularly by minimal invasive advanced hemodynamic monitoring, for example, by using FloTrac® technology. However, the available evidence remains limited and is currently based on cohort studies for radical cystectomy.^9^, ^10^, ^11^

The present study aims to expand the current evidence base regarding the efficacy of intraoperative FloTrac® monitoring in reducing paralytic ileus and to identify further predictive factors for its occurrence in a large, single‐centre patient cohort.

## 2 PATIENTS AND METHODS

### 2.1 Patients

The study cohort comprised 383 patients who underwent radical cystectomy at the Department of Urology, University Hospital of Cologne, between 2018 and 2024. A total of 87 patients were excluded due to the creation of ureterocutaneostomies or owing to insufficient reconstructability of clinical data. Consequently, 296 patients were included in the final retrospective analysis. The indication for cystectomy was predominantly oncological, most commonly due to urothelial carcinoma, and less frequently because of locally advanced prostate carcinoma.

### 2.2 Definition of paralytic ileus

A paralytic ileus was defined as the postoperative placement of a nasogastric tube, the occurrence of postoperative vomiting and/or the inability to tolerate solid food intake beyond 7 days after surgery. This definition is consistent with previously published literature.^12^ In cases of suspected paralytic ileus, patients receive 50–100 mL of oral contrast agent followed by a radiographic gastrointestinal transit study. In the presence of clinical symptoms such as vomiting, a nasogastric tube is inserted.

### 2.3 Perioperative protocol

No preoperative bowel preparation was performed. Patients were advised to follow a high‐calorie diet during the week preceding radical cystectomy. Overall, our Enhanced Recovery After Surgery (ERAS) protocol aims to integrate several perioperative measures that have previously been associated with improved postoperative recovery. These measures include structured preoperative counselling, routine placement of abdominal drainage, immediate postoperative removal of the nasogastric tube and allowance of oral fluid intake in the early postoperative period.

Perioperatively, patients routinely receive a cephalosporin in combination with metronidazole as antibiotic prophylaxis. In the FloTrac® group, a radial arterial line (20 G) was placed for continuous invasive blood pressure monitoring following induction of anaesthesia. The FloTrac® sensor was connected to the arterial line and interfaced with the Vigileo™ monitor, third generation (Edwards Lifesciences, Irvine, CA, USA). Baseline advanced hemodynamic monitoring parameters were recorded, and patients were subsequently monitored continuously with FloTrac® in addition to standard intraoperative monitoring.

Cystectomy was performed either via an open approach through a lower midline laparotomy or as a robot‐assisted procedure with intracorporeal urinary diversion. In cases with an oncologic indication, an extended pelvic lymph node dissection was systematically performed, encompassing the obturator fossa as well as the internal and external iliac regions up to the level of the aortic bifurcation.

All bowel anastomoses were created using a standardized side‐to‐side stapling technique, employing either a 60‐ or 80‐mm gastrointestinal anastomosis stapler in combination. In our standard practice, bowel anastomoses are not constructed by hand‐sewing techniques. For urinary diversion, an ileal segment of approximately 10–15 cm was utilized for ileal conduit formation, whereas 50–70 cm of ileum was employed for orthotopic neobladder or continent pouch reconstruction.

At our institution, all patients received parenteral nutrition until the resumption of bowel function. Once flatulence or bowel movements were observed, enteral nutrition was gradually reintroduced, and parenteral support was discontinued. Emphasis was placed on early postoperative mobilization. Routine admission to a postoperative intensive care unit was not standard practice and was reserved only for cases involving significant comorbidities. Postoperative management also included retrograde ureteral catheter imaging on postoperative day seven. In patients undergoing neobladder or pouch reconstruction, a cystographic assessment was additionally performed between postoperative Days 10 and 12.

### 2.4 Statistics

Descriptive statistics were reported as mean (M) and standard deviation (SD). For normally distributed variables, comparisons were done using Student's *t*‐test. Non‐normally distributed continuous variables were analysed with the Mann–Whitney *U* test and categorical variables with the chi‐squared test. Survival analyses were conducted using the log‐rank test.

Predictive factors for paralytic ileus were assessed by univariable logistic regression, reporting the regression coefficient (*β*), standard error (SE), *Z*‐statistic, *p*‐value and odds ratio (OR; exp(β)). A multivariable logistic regression model was subsequently fitted using backward elimination based on the Wald statistic. Variables were stepwise removed until further exclusion no longer improved model performance, as measured by OR and model fit indices.

Descriptive and inferential statistical analyses were performed using Python (version 3.12; Python Software Foundation, Wilmington, DE, USA) with the statistical packages *statsmodels* (version 0.14.4) and *scipy* (version 1.14.1).

### 2.5 Ethics

This retrospective investigation adhered to the ethical principles of the Declaration of Helsinki and received approval from the institutional ethics committee of cologne (reference number 25–1181‐retro).

## 3 RESULTS

### 3.1 Baseline characteristics

The study cohort comprised 233 (78.7%) male and 63 (21.3%) female patients aged between 18 and 88 years (mean = 66.3 years; SD = 12.35) (Table 1). At the time of inclusion, most patients were classified as ASA Grade 2 (*n* = 144; 48.6%) or Grade 3 (*n* = 140; 47.3%), with a small proportion (<5%) presenting with either lower (ASA grade 1) or higher (ASA grade 4) scores. A total of 112 patients (37.8%) were smokers, and 41 (13.9%) had diabetes mellitus.

**TABLE 1:** Baseline characteristics.

Variable	Distribution
Age	66.3 ± 12.34
ASA	
1	6 (2%)
2	144 (48.6%)
3	140 (47.3%)
4	6 (2.0%)
Sex	
Male	233 (78.7%)
Female	63 (21.3%)
Smoker	112 (37.8%)
Diabetes	41 (13.9%)
BMI	26.9 ± 10.31
Previous radiotherapy of the pelvis	51 (17.2%)
Surgical indication	
Urothelial carcinoma	214 (72.3%)
NMIBC	16/214 (7.4%)
Prostate cancer	53 (17.9%)
Other	29 (9.8%)
Urinary diversion	
Neobladder	47 (15.9%)
Ileal conduit	239 (80.7%)
Pouch	10 (3.4%)
Neoadjuvant chemotherapy	122/198 (61.61%)
Hydronephrosis prior surgery	95 (31.9%)
Bacterial load in urine culture	98 (33.1%)

*Note*: The cohort included 296 patients (233 men, 63 women; mean age 66.3 ± 12.4 years). Most were classified as ASA II (48.6%) or III (47.3%); 37.8% were smokers, and 13.9% had diabetes. The primary indication was urothelial carcinoma (72%), followed by prostate cancer (17.9%) and other malignancies (9.8%). Among urothelial carcinomas, 7.4% were therapy‐refractory NMIBC; 61.1% of muscle‐invasive cases received neoadjuvant chemotherapy. Neoadjuvant chemotherapy is the preferred treatment prior to upfront radical cystectomy in all patients eligible for cisplatin according to the classical Galsky criteria. At our institution, cisplatin eligibility is extended to selected patients with a glomerular filtration rate of 40–60 mL/min using a split‐dose regimen. Preoperative urine cultures showed bacterial growth in 33.1% of patients.

The most frequent indication for surgery was urothelial carcinoma (72%), followed by symptomatic or locally advanced prostate cancer (17.9%). Less common etiologies (9.8%) included pure squamous cell carcinoma of the bladder, locally advanced endometrial or vulvar carcinoma. Among patients with urothelial carcinoma, 16 of 214 cases (7.4%) were NMIBC. Of those with MIBC (*n* = 198), 61.1% received neoadjuvant chemotherapy.

As part of the standardized preoperative assessment at our institution, a urine culture was routinely performed; bacterial growth was detected in 33.1% of samples.

### 3.2 Predictors of postoperative paralytic ileus

To identify variables associated with the development of postoperative paralytic ileus, a multivariable logistic regression analysis was conducted. In the initial model (Model 0), all available parameters—including patient demographics, medical history, preoperative laboratory values, intraoperative data and immediate postoperative variables—were entered as potential predictors of paralytic ileus. The results of this comprehensive model are summarized in Table 2.

**TABLE 2:** Predictors of postoperative paralytic ileus.

Variable	OR	95% CI	*p*‐value	OR	95% CI	*p*‐value
ASA (ASA 1)	1.44	(0.81; 2.58)	0.215			
Age	1.00	[0.96; 1.03]	0.760			
Sex (female)	5.21	[1.92; 14.11]	**0.001**	3.78	[1.83; 7.81]	**<0.001**
Diabetes	0.74	[0.31; 1.76]	0.501			
Previous history of cardiac events						
Myocardial infarction	0.53	[0.08; 3.44]	0.498			
Heart failure	1.92	[0.63; 5.84]	0.248			
Coronary heart disease	1.03	[0.35; 3.02]	0.952			
Medication						
Marcumar	0.96	[0.07; 12.97]	0.978			
NOAK	0.48	[0.14; 1.63]	0.242			
Metformin	0.98	[0.22; 4.46]	0.982			
Smoker	0.83	[0.43; 1.58]	0.572			
Weight	1.05	[1.01; 1.10]	**0.029**	1.04	[1.00; 1.08]	**0.034**
BMI	0.88	[0.75; 1.02]	0.088	0.90	[0.79; 1.02]	0.101
NMIBC	0.87	[0.29; 2.60]	0.800			
Intravesical therapy (BCG)	0.31	[0.01; 19.26]	0.589			
Radiotherapy	0.74	[0.33; 1.68]	0.477			
Hydronephrosis	0.62	[0.32; 1.16]	0.135	0.74	[0.44; 1.23]	0.239
Abominal surgery	1.18	[0.61; 2.28]	0.629			
Bacterial colonization (preop urine)	0.89	[0.48; 1.65]	0.703			
Neoadjuvant chemotherapy	1.42	[0.67; 3.00]	0.357			
Haemoglobin	1.55	[0.83; 2.90]	0.171			
Haematocrit	0.87	[0.71; 1.08]	0.216			
Platelets	1.00	[1.00; 1.00]	0.708			
LDH	1.00	[0.99; 1.00]	0.147	1.00	[0.99; 1.00]	0.109
Albumin	0.94	[0.86; 1.02]	0.125	0.95	[0.90; 1.01]	0.092
Fibrinogen	0.98	[0.73; 1.32]	0.879			
Creatinine	1.04	[0.94; 1.13]	0.461			
CRP	1.01	[0.99; 1.03]	0.119			
Surgical indication Prostate cancer	1.07	[0.43; 2.64]	0.887			
Surgical approach Robotic	0.14	[0.02; 1.01]	0.051	0.17	[0.03; 0.89]	**0** **.036**
Urinary diversion Conduit	0.68	[0.29; 1.59]	0.369			
Operation time	1.00	[1.00; 1.01]	0.592			
Blood loss	1.00	[1.00; 1.00]	0.481			
Peridural catheter	1.21	[0.57; 2.57]	0.611			
Crystalloid solution	1.00	[1.00; 1.00]	0.564			
Colloid solution	1.00	[1.00; 1.00]	0.313			
RBC units	1.00	[1.00; 1.00]	0.871			
Mean arterial pressure < 65 min	1.04	[0.89; 1.21]	0.630			
Mean arterial pressure < 60 min	0.41	[0.00; >1000.00]	0.892			
FloTrac	0.37	[0.16; 0.88]	**0.024**	0.37	[0.18; 0.78]	**0.008**
Opioid administration	1.44	[0.67; 3.10]	0.350			
ICU stay	0.81	[0.42; 1.57]	0.533			

*Note:* Statistically significant p‐values (*p* < 0.05) are presented in bold.

**FIGURE 1:**
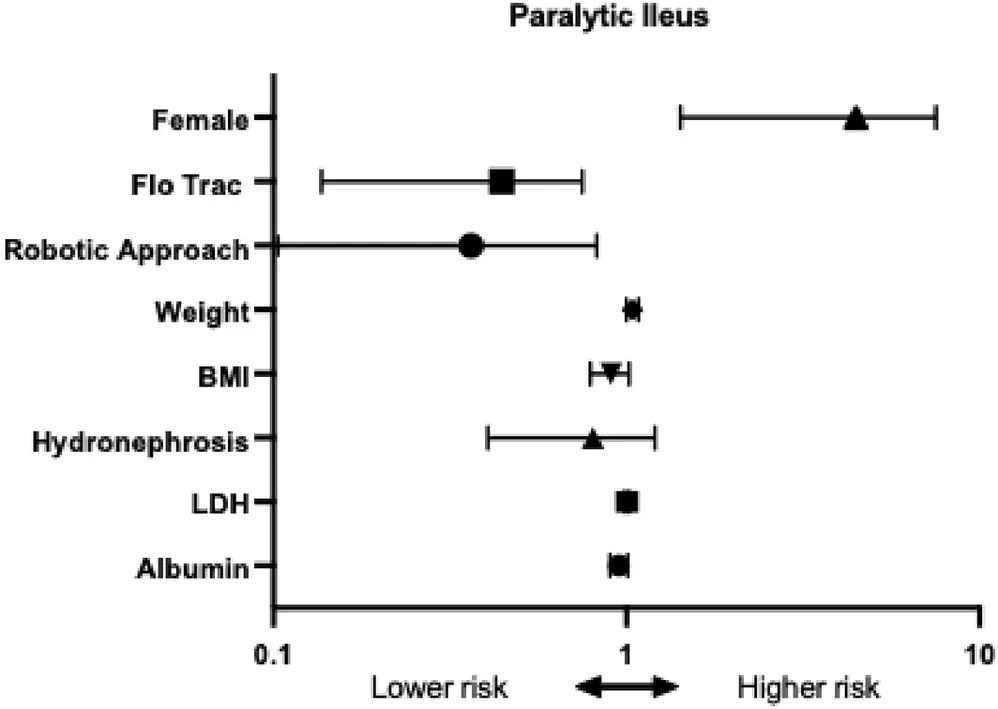
Forest plot of the multivariable regression analysis for predictors of postoperative paralytic ileus. Odds ratios with 95% confidence intervals are shown; values left of 1 indicate lower risk and values right of 1 indicate higher risk. Variables included sex, FloTrac monitoring, robotic approach, weight, BMI, hydronephrosis, LDH and albumin.

In Model 0, FloTrac® monitoring, sex and body weight emerged as statistically significant predictors. Subsequent stepwise model reduction eliminated 31 of the original 39 variables without compromising predictive accuracy, as indicated by stable or improved estimates for relative risk (RR) and odds ratio (OR). The final optimized model (Model 1) retained nine predictors, remaining within the recommended parameter limits (Figure 1). This model demonstrated enhanced predictive performance, with an RR of 1.85 (95% CI: 1.02–3.35) and an OR of 3.77 (95% CI: 2.08–6.82)—both statistically significant, as their confidence intervals excluded unity. In Model 1 (see Table 3), FloTrac® monitoring, sex, body weight and surgical approach were identified as significant independent predictors, underscoring their prognostic relevance. Notably, the surgical approach (robotic vs. open) independently predicted the occurrence of paralytic ileus, with robotic surgery being associated with a lower risk.

**TABLE 3:** Differences between the groups with and without FloTrac monitoring.

Variable	FloTrac (*n* = 49)	Without FloTrac (*n* = 247)	*p*‐value
Approach	Open 47 (95.6%)	Open 236 (95.5%)	0.791^a^
	Robotic 2 (4.1%)	Robotic 11 (4.5%)	
Surgical length (in min)	284.4 ± 95.25	287.5 ± 73.52	0.826^b^
Blood loss (in mL)	379.6 ± 208.65	481.0 ± 558.96	**0.030** ^b^
Cristalloid Solution (in mL)	3173.5 ± 1602.57	3360.3 ± 1378.27	0.449^b^
Colloid solution (in mL)	0.0 ± 0.0	32.4 ± 159.28	**0.002** ^b^
Postoperative haemoglobin (in g/dL)	9.6 ± 1.61	9.5 ± 1.62	0.485^c^
Postoperative haematocrit (in g/dL)	29.0 ± 4.89	28.5 ± 4.62	0.871^c^
Postoperative albumin (in g/dL)	29.4 ± 3.23	28.2 ± 4.28	0.214^c^
Postoperative creatinine (in mg/dL)	1.2 ± 0.39	1.3 ± 0.79	0.613^c^
Postoperative CRP (in g/dL)	82.8 ± 43.35	89.0 ± 53.14	0.985^c^
Clavien Dindo complications			
3	7 (14.3%)	41 (16.6%)	0.171^a^
4	0	3 (1.2%)	
5	0	3 (1.2%)	

*Note*: Differences between groups were analysed using the chi‐square test for categorical variables and the *t*‐test for continuous variables. For the ANCOVA, preoperative laboratory values were included as covariates in the statistical analysis. Statistically significant p‐values (*p* < 0.05) are presented in bold.

^a^ Chi‐squared test.

^b^ Students *t*‐test.

^c^ ANCOVA test.

Although BMI remained part of the final model, it did not reach statistical significance (*p* = 0.101), indicating a trend‐level association. Conversely, hydronephrosis, urinary diversion and the preoperative laboratory parameters lactate dehydrogenase (LDH) and albumin were not significant on their own but appeared to exert moderating effects. Excluding these variables during model refinement reduced both RR and OR values—rendering the RR nonsignificant—suggesting that these factors contribute indirectly to the overall robustness and explanatory capacity of the model.

### 3.3 Differences between FloTrac®

A significant difference was observed in intraoperative blood loss, with patients in the FloTrac® group exhibiting a markedly lower mean blood loss of 379.6 ± 208.7 mL compared to those in the control group without FloTrac® monitoring (481.0 ± 558.96 mL; *p* = 0.030). Furthermore, a lower volume of colloid fluids was administered in the control group without FloTrac® monitoring (*p* = 0.002). All other perioperative parameters showed no statistically significant differences between the two groups, including postoperative complications, which were generally low in incidence.

(*p* ≥ 0.05, Table 3).

## 4 DISCUSSION

Paralytic ileus is a common complication following radical cystectomy, occurring in approximately 20%–30% of patients and potentially precipitating additional postoperative morbidity. We conducted a retrospective cohort study involving 296 patients to identify potential predictors of paralytic ileus. Our analysis demonstrated that female sex and higher body weight were associated with an increased risk of developing this complication. In contrast, the use of FloTrac® advanced hemodynamic monitoring was linked to a significantly reduced risk according to our data.

Initial studies have already examined goal‐directed hemodynamic therapy in the context of radical cystectomy. In a retrospective cohort, Ghoreifi et al. demonstrated that although intraoperative fluid administration could be reduced, this did not translate into a lower rate of postoperative complications.^10^ A prospective study focusing on overall postoperative morbidity reached similar conclusions: The rate of general complications was 72% in the FloTrac® group compared with 83% in the conventional fluid management group. However, this study was limited by a relatively small sample size, leaving open the question of whether a larger cohort might have resulted in statistical significance. Notably, no explicit data on paralytic ileus were reported.^11^ Another prospective study with a larger cohort found no reduction in the incidence of paralytic ileus under FloTrac®. Overall complication rates were likewise comparable between groups, with the exception of acute kidney injury, which occurred more frequently in the FloTrac® cohort.^9^ A potential explanation for the observed findings may lie in the study design. In prospective randomized trials, all participating personnel—in this case, the anesthesiologists—are aware that fluid management represents a central focus of the investigation. This awareness, irrespective of group allocation, may lead the entire team to perform in a particularly attentive and ‘optimal’ manner, simply because they know they are being observed (Hawthorne effect). Although the blinding procedure implemented in the study may mitigate this phenomenon, it cannot fully eliminate it. Experienced anesthesiologists in the control group may rely more strongly on their clinical judgement and individualized volume management, which could reduce the perceived need for additional advanced hemodynamic monitoring tools. In this context, it is worth emphasizing the importance of avoiding excessive intraoperative fluid administration.^13^ As a result, intergroup differences within the controlled study setting are likely to be attenuated. In routine clinical practice outside of such trials—as in the present retrospective analysis—decisions regarding the type and extent of volume therapy are more likely influenced by individual habits, subjective assessments, and everyday clinical workload. Under these circumstances, the use of a technical monitoring system such as FloTrac® may indeed facilitate more precise fluid management and thereby yield a more pronounced clinical effect. Nevertheless, it is important to emphasize that our data did not reveal any difference in the total amount of intraoperative fluid administered between groups and that the observed effect may therefore be attributable to advanced hemodynamic monitoring and treatment parameters which were not registered in our study.

Beyond urologic surgery, multicentre prospective trials have shown that FloTrac® can reduce general postoperative complications, although these studies included gynecologic, urologic and visceral surgical procedures collectively.^14^ In our study, the overall rate of complications did not differ between groups.

Furthermore, our data indicate a statistically higher risk of paralytic ileus with increasing body weight. These findings are consistent with a retrospective study by Svatek et al., which similarly reported a higher incidence of postoperative ileus in overweight patients.^15^ More broadly, previous research has demonstrated that obesity is associated with an increased rate of complications following radical cystectomy. The fact that BMI itself did not reach statistical significance in our analysis and only demonstrated a trend may be explained by the well‐described association between both severe underweight and postoperative complications, thereby diluting a linear BMI effect.^16^

In our multivariable regression analysis, robotic‐assisted cystectomy was associated with a lower risk of postoperative paralytic ileus. However, only 4% of patients in our cohort underwent robotic surgery; extrapolation to broader clinical practice should therefore be cautious. Large Phase III trials have demonstrated comparable ileus rates between open and robotic approaches. In the RAZOR trial, postoperative ileus occurred in 20% after open cystectomy and 22% after robotic‐assisted cystectomy.^17^ Taken together, these data suggest that small‐bowel mobilization during surgery does not inherently increase ileus risk; other perioperative factors likely play a more decisive role.

Our data demonstrate that women have a significantly increased risk of developing postoperative paralytic ileus. Female patients exhibited more than twice the likelihood of experiencing this complication compared with male patients. The question of causality, however, remains unresolved. In colorectal surgery, paralytic ileus has been reported to occur more frequently in men, whereas other studies have found no sex‐specific differences—although these analyses may have been influenced by the slightly lower proportion of women included (21% vs. 16%).^15^ A potential explanation may lie in the higher prevalence of constipation among women, which itself has been identified as an independent risk factor for paralytic ileus.^18^, ^19^

Our study is, by design, not without limitations. First, it is retrospective and single‐centre in nature. However, as discussed above, the retrospective approach may also be considered a strength, as it allows for the evaluation of real‐world data in a standardized clinical setting.

Moreover, the number of patients monitored using the FloTrac® system was relatively small, and even fewer underwent robotic‐assisted cystectomy. Consequently, the interpretation and clinical translation of findings related to these subgroups should be approached with caution. Nevertheless, with 383 patients screened and 296 included in the final analysis, our cohort represents a considerable sample size for this type of investigation.

Furthermore, specific advanced hemodynamic monitoring and treatment parameters that could potentially explain the effect of FloTrac® monitoring on the incidence of postoperative paralytic ileus were not identified. Considering the complex relationship between advanced hemodynamic monitoring parameters and their influence on hemodynamic therapy management, it is possible that additional perioperative factors not assessed in the present study may also contribute to this effect. In particular, differences in intraoperative catecholamine administration may represent a relevant confounder. Higher vasopressor requirements could potentially impair splanchnic perfusion and intestinal motility, thereby contributing to the development of postoperative paralytic ileus. However, intraoperative catecholamine doses were not systematically recorded in our cohort and therefore could not be incorporated into the present analysis. Further studies are therefore warranted to clarify which FloTrac® parameters, and resulting therapeutic decisions could potentially explain its influence on the incidence of paralytic ileus.

## 5 CONCLUSIONS

Overall, we believe that advanced haemodynamic monitoring such as FloTrac® monitoring represents a simple, cost‐effective tool that can support intraoperative hemodynamic management and may have the potential to reduce the incidence of postoperative paralytic ileus. However, our data do not suggest a reduction in the overall postoperative complication rates. Independent of FloTrac® use, strict adherence to ERAS protocols remains particularly important in female patients and in those with higher body weight.

## AUTHOR CONTRIBUTIONS

All authors contributed to the conception, drafting, critical revision, and final approval of the manuscript.

## CONFLICT OF INTEREST STATEMENT

Constantin Rieger: Honoraria: Medac Germany, Astellas. Advisory Board: Pfizer. David Pfister: Consulting Fees: MSD. Travel: Bayer, Janssen. Advisory Board: BMS, Janssen, MSD, Pfizer. Honoraria: Bayer, Pfizer, Janssen, AstraZeneca, MSD, BMS. Axel Heidenreich: Honoraria: Amgen, Astellas, AstraZeneca, Bayer, BMS, Clovis Oncology, Janssen, Pfizer, Takeda. Advisory Board: Astellas, Bayer, Janssen, MMS. Research Grant: BMS. The remaining authors have no disclosures.
